# Failure of the Pipeline Embolization Device in Posterior Communicating Artery Aneurysms Associated with a Fetal Posterior Cerebral Artery

**DOI:** 10.1155/2016/4691275

**Published:** 2016-03-16

**Authors:** Mario Zanaty, Nohra Chalouhi, Robert M. Starke, Pascal Jabbour, Katherine O. Ryken, Ketan R. Bulsara, David Hasan

**Affiliations:** ^1^Department of Neurosurgery, University of Iowa Hospital and Clinics, 200 Hawkins Drive, Iowa City, IA 52242, USA; ^2^Department of Neurological Surgery, Thomas Jefferson University and Jefferson Hospital for Neuroscience, 901 Walnut Street, 3rd floor, Philadelphia, PA 19107, USA; ^3^Department of Neurosurgery, University of Virginia, Charlottesville, VA 22908, USA; ^4^Department of Neurosurgery, Yale University, New Haven, CT 06519, USA

## Abstract

The pipeline embolization device has emerged as an important endovascular option. This is in part due to safety, efficacy, and possibly the ability to shorten the operative time considerably. With this new technology, some limitations are emerging as experience accumulates. We report three cases of posterior communicating (PCOM) artery aneurysms associated with fetal posterior cerebral artery where pipeline embolization was unsuccessful in obliterating the aneurysms. PCOM artery aneurysms associated with a fetal PCA should be managed either by microsurgical clipping or coiling when feasible.

## 1. Background and Importance

Endovascular therapy is, at some institutions, the first-line treatment for intracranial aneurysms (IAs) for its efficacy and safety profile [1]. The use of the pipeline embolization device (PED) has recently expanded to cover many types of IAs in various locations [2, 3]. In some institutions, the PED is even used for treatment of morphologically complex aneurysms in the setting of subarachnoid hemorrhage [1, 4]. Posterior communicating artery aneurysms (PCOM) associated with a fetal posterior cerebral artery (PCA) in general present a challenge to coiling due to the fact that most often the origin of the fetal PCA arises from the aneurysm side wall. The hope was that, with flow diversion, these aneurysms would progress to occlusion while the PCOM artery would remain patent due to physiologic demand. This is the first study to evaluate the use of flow diversion in PCOM artery aneurysms PCOM artery aneurysmswith a fetal PCA.

## 2. Clinical Presentation

### 2.1. Case 1

**Figure 1:**
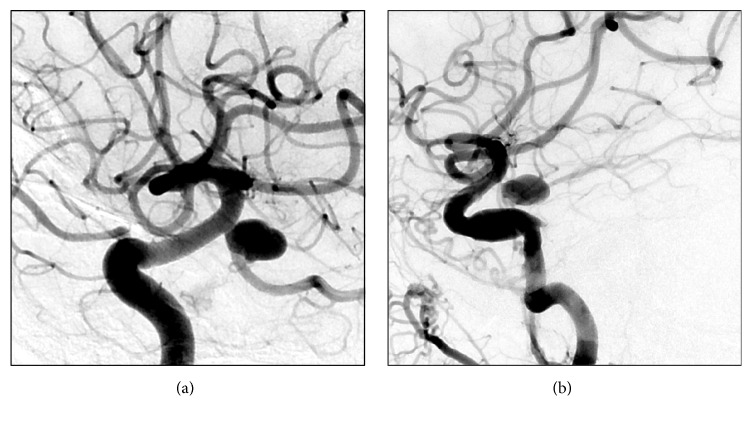
Illustrative case. (a) Preop angiogram of the same patient showing the large PCOM aneurysm. (b) 3-month follow-up angiogram showing that the PCOM aneurysm remains patent despite pipeline stenting.

The first patient is a 67-year-old female who presented with Hunt and Hess grade (HHG) III subarachnoid hemorrhage. A right-sided external ventricular drain was placed followed by catheter angiogram that demonstrated a left PCOM aneurysm 14 × 10 mm with a fetal PCA. The fetal PCA arose from the sidewall of the aneurysm. We decided to proceed with endovascular intervention to secure the aneurysm ultraearly since the patient has a high HHG. An attempt to secure the aneurysm with stent-assisted coiling was unsuccessful. The decision was made to secure the aneurysm with a PED. A single PED was successfully deployed across the neck of the aneurysm. The patient made an excellent recovery and was discharged home after adequate hospital stay. She was placed on aspirin 325 mg daily, Plavix 75 mg daily. Follow-up angiogram at 3 months (Figure 1) demonstrated a persistent full sized PCOM aneurysm with sluggish filling. The patient was admitted and underwent successful microsurgical clipping. She has now a Karnofsky performance status of 60.

### 2.2. Case 2

The second patient is a 57-year-old male who was incidentally found to have a cerebral aneurysm during a work-up for his headaches. Given his severe comorbidities, we decided to proceed with an endovascular intervention. The pipeline stent has an established efficacy in supraclinoid aneurysm with less recurrence when compared with coiling [1]. We typically place the pipeline device under monitored anesthesia to monitor for complications [5]. For all these reasons, the patient underwent an elective PED placement for a 10 × 7 mm right-sided fetal PCOM aneurysm with a 4 mm neck and a fetal PCA. He was placed on aspirin 325 mg and Plavix 75 mg daily. The 6-month follow-up angiogram demonstrated failure of the treatment with a persistent filling and flow into the aneurysm. The patient's aneurysm was successfully treated with microsurgical clipping. His KP is 100.

### 2.3. Case 3

The third patient is a 68-year-old male who presented with a right parieto-occipital stroke. During the work-up he was found to have an incidental right fetal PCA with a PCOM aneurysm. He had severe comorbidities and was deemed high risk for anesthesia. He underwent pipeline embolization under monitored anesthesia. 6-month follow-up showed that the aneurysm continues to be filled. His aneurysm was eventually treated with microsurgical clipping on an elective basis, after sufficient recovery from the stroke. The procedure was without major complications. He continues to have a KPS of 70 due to his previous stroke. In all three cases, one pipeline was sufficient for complete neck coverage.

## 3. Discussion

A fetal PCA variant is defined as a PCOM artery with a caliber larger that the P1 segment of the PCA with no filling of the PCA via the P1 segment. Its incidence is estimated between 4 and 29% of patients and bilateral fetal PCOM variants occur in 1–9% of patients [6, 7]. Because fetal PCOM arteries are the only supply to the PCA, care must be taken not to compromise flow to this artery during clipping or coiling of PCOM artery aneurysms [8]. The aneurysm neck originates from the PCOM artery in 0.1–2.8% of all aneurysms and 4.6–13% of PCOM artery aneurysms [8, 9]. These aneurysms are encountered mostly with large or fetal PCOM arteries and pose a challenge to coiling and clipping due to the carefulness required to maintain the flow through the PCOM [9]. With fetal PCA, it is necessary to have an optimal dome to neck ratio for safe coiling. For these reasons, surgeons are classically more inclined to clip rather than coil PCOM artery aneurysms with fetal PCA. One notable example would be the study by Zada et al. [10], who evaluated the treatment of 30 PCOM artery aneurysms with a fetal PCA origin and reported that only 5 underwent coil embolization while the others underwent open surgical treatment.

On the other hand, there is solid data to support endovascular management of cerebral aneurysms, including PCOM arteries in general. The ISAT trial [11] showed less morbidity/mortality and more independence at 1 year in patient harboring ICA aneurysms who underwent endovascular versus surgical treatment. However, endovascular coiling has a higher recurrence rate, especially when the neck is unfavorable. Raymond et al. reported a recurrence rate of 33.6% with a retreatment rate of 20.7% after coiling and found that PCOM aneurysm had the second highest recurrence rate at 37.2% [12].

Flow diversion relies on a concept of endoluminal reconstruction of the parent artery and the aneurysm neck by excluding the aneurysm from the circulation, therefore avoiding the risks endosaccular coiling. The stasis of blood flow in the aneurysm leads to an inflammatory response followed by thrombosis and “healing” of the aneurysm while the stent acts as a scaffold for neointimal proliferation and remodeling of the parent vessel. Flow diversion has provided a means to treat large, giant, and morphologically complex aneurysms with similar or lesser combined morbidity and mortality [13, 14], and better or similar outcomes [15, 16].

**Figure 2:**
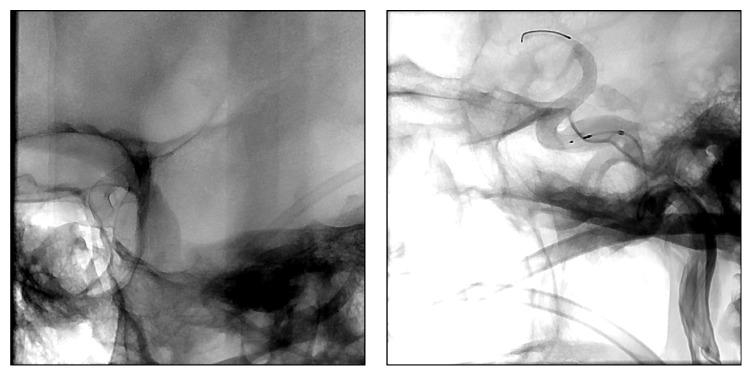
Unsubtracted X-ray showing the relation of the pipeline to the skull base and the vasculature at the circle of Willis.

In the present report, we highlight for the first time an important limitation of flow diversion in PCOM artery aneurysms with a fetal PCA. The treatment rationale was to pursue endovascular treatment in the patients, due to a high HHS in one patient and the severe comorbidities and the high risk of anesthesia in the others. As for the type of endovascular intervention, there is an institutional bias in the literature. The PED has been reported from many institutions to have an excellent response and less recurrence with coiling for supraclinoid aneurysms [1], and our plan was to expand the indication. We place the pipeline under monitored anesthesia so we can monitor complications [5]. This is very important when there are perforators at risk. However, our treatment rational failed, and hence we are converting back to coiling or clipping for fetal PCAs. As for positioning of the device, we placed it in the ICA in an attempt to divert the high flow coming from the ICA to the PCOM and into the PCA (Figure 2). However, this theory failed in practice and we are converting back to clipping or coiling for these aneurysms. We did not feel that placing the pipeline in the PCA would help since the high flow is coming from the ICA.

In all three patients, flow diversion failed to promote any aneurysm occlusion. This is due to the fact that flow through the fetal PCA and the aneurysm sac remains high even after placement of flow diverters due to the high physiologic demand. This stands in contrast to other locations such as the ophthalmic artery or even nonfetal PCOM arteries where the PED is able to drive aneurysm occlusion while preserving the patency of side branches. Coiling was not considered due to sidewall perforators, which are preserved with flow diversion due to increased demand and vasodilation in the vascular bed supplied by the perforators. In our institution, aspirin and Plavix are given for a total of 6 weeks after which Plavix is stopped. Patients underwent elective clipping while on aspirin. Based on this report, we recommend that PCOM artery aneurysms with a fetal PCA be treated with microsurgical clipping. Attempting flow diversion in these cases will unnecessarily expose the patient to additional procedural risk and make surgical clipping even more technically complex. Moreover, holding antiplatelet therapy for surgical clipping increases the risk of construct thrombosis.

In their review of the literature, Golshani et al. [8] found that PCOM artery aneurysms with an elongated fundus, true PCOM artery aneurysms, and aneurysms associated with a fetal posterior communicating artery may have better outcome with surgical clipping in terms of completeness of occlusion and preservation of the posterior communicating artery. The authors concluded that as endovascular technology improves, endovascular treatment of PCOM artery aneurysms might become equivalent or preferable in the near future. This premise failed to hold in our series.

## 4. Conclusion

Although flow diversion is an appealing option for the management of supraclinoid aneurysms, it does not appear to be a good treatment modality for PCOM artery aneurysms with a fetal PCA. These aneurysms should be managed primarily with microsurgical clipping or coiling when feasible.
